# User Characteristic Aware Participant Selection for Mobile Crowdsensing

**DOI:** 10.3390/s18113959

**Published:** 2018-11-15

**Authors:** Dapeng Wu, Haopeng Li, Ruyan Wang

**Affiliations:** 1 School of Communication and Information Engineering, Chongqing University of Posts and Telecommunications, Chongqing 400065, China; s160131070@stu.cqupt.edu.cn (H.L.); wangry@cqupt.edu.cn (R.W.); 2 Key Laboratory of Optical Communication and Networks, Chongqing 400065, China; 3 Key Laboratory of Ubiquitous Sensing and Networking, Chongqing 400065, China

**Keywords:** mobile crowdsensing, participant selection, regional heat, user characteristic

## Abstract

Mobile crowdsensing (MCS) is a promising sensing paradigm that leverages diverse embedded sensors in massive mobile devices. One of its main challenges is to effectively select participants to perform multiple sensing tasks, so that sufficient and reliable data is collected to implement various MCS services. Participant selection should consider the limited budget, the different tasks locations, and deadlines. This selection becomes even more challenging when the MCS tries to efficiently accomplish tasks under different heat regions and collect high-credibility data. In this paper, we propose a user characteristics aware participant selection (UCPS) mechanism to improve the credibility of task data in the sparse user region acquired by the platform and to reduce the task failure rate. First, we estimate the regional heat according to the number of active users, average residence time of users and history of regional sensing tasks, and then we divide urban space into high-heat and low-heat regions. Second, the user state information and sensing task records are combined to calculate the willingness, reputation and activity of users. Finally, the above four factors are comprehensively considered to reasonably select the task participants for different heat regions. We also propose task queuing strategies and community assistance strategies to ensure task allocation rates and task completion rates. The evaluation results show that our mechanism can significantly improve the overall data quality and complete sensing tasks of low-heat regions in a timely and reliable manner.

## 1. Introduction

With the rapid development of wireless communications and the explosive popularity of mobile devices, the combination of mobile sensing, distributed computing, and crowd-sourcing has promoted the Mobile Crowdsensing (MCS) paradigm [1,2]. Mobile devices serve as basic sensing units and a crowd of them form a large-scale sensing network, used for completing some complex sensing tasks that are impossible for individuals [3]. Due to the low deploying cost and high sensing coverage, this new sensing paradigm has been supporting a broad range of applications, such as map physical spatial fields [4], smart transportation [5], environmental monitoring [6] and digital map [7]. In a general multi-task MCS system, each sensing task is first initiated and announced by a task planner (task owner) via a service platform. Then, the task is assigned to a set of participants, who are selected from a pool of mobile users. Participants use their devices to complete the corresponding sensing tasks, and then upload the sensing data to the service platform through a mobile cellular network or short-range wireless communication (e.g., Bluetooth or Wi-Fi) [8,9,10,11].

In MCS applications, the service platform selects participants to complete sensing tasks. Because the user candidate pool can be rather large, the service platform should allocate several tasks to selected participants. Thus, participant selection becomes one of the main challenges in MCS [12,13,14]. In fact, there are always limited budgets and time when the service platform selects participants [15,16,17,18]. At the same time, the service platform needs to have enough users to participate in sensing tasks. The user distribution in urban areas obeys independent Poisson Scattering, or by scattering with repulsion [19,20] or attraction [21,22], resulting in some so-called high heat with massive users at a certain moment, where multiple users compete for the same task at the same time with a certain probability. However, in other regions, the user arriving interval cannot be determined, and the task completion time is uncertain. In addition, after the users are assigned sensing tasks, task failures may occur due to the changes in environmental conditions, and it is difficult to guarantee the quality of the data submitted by selfish mobile users [23]. Therefore, designing an efficient participant selection strategy is necessary.

For user-centric MCS networks, users have obvious social characteristics, and their willingness to participate will affect the task performance and number of enough participants to complete a sensing task [24]. In addition, the user reputation is closely related to the reliability of sensing data and evaluated by a reasonably designed model [25]. After receiving sensing tasks, high reputation users can delegate the tasks to other users and still get paid, resulting in unreliable sensing data [26,27]. User activity also has a great impact on the task execution performance of the service platform. Users with higher regional activity are more likely to visit more locations and thus able to accomplish more sensing tasks [28,29]. Moreover, users interact with others from different communities to form a sensing network covering the real-world communities. The users with a high level of social activity have a wide covering range, are more stable and reliable, and can complete more sensing tasks. Hence, the MCS participant selection process should fully consider the user characteristics and the difference of the regional heat.

In this paper, we propose the UCPS mechanism to improve the credibility of task data in sparsely populated regions and to reduce the task failure rate under limited budgets and time. Our main results and key contributions are summarized as follows:

(1) First, we evaluate the heat of different regions in the MCS service scenario based on the number of active users, their average residence time, and sensing tasks history. Then, the user state information and sensing task records are combined to calculate the willingness, reputation and activity of users, respectively. Furthermore, we analyze the influence of user characteristics on the probability of completing sensing tasks, credibility of the submitted task data and ability of participants to complete the task.
(2) Second, we design a task queuing strategy and a community assistance strategy. According to users’ activity and willingness, the upper limit of queues is dynamically set. The participants complete the tasks in the queue according to their priority. When a sensing task cannot be performed by a participant due to the changes of the participants’ own conditions, it can be assisted by the community to reduces the task failure rate. In addition, our designed community assistance strategy attracts users to participate in the sensing tasks extensively and further expands the MCS coverage.
(3) Finally, we propose UCPS-H and UCPS-L algorithms for high-heat and low-heat regions, respectively. In the high-heat regions, we evaluate the comprehensive data quality by leveraging user characteristics and task bidding, and then select participants for the maximum task data quality. In the low-heat regions, we divide the participant selection process into multiple stages. Within each stage, participants with reliable profits exceeding the dynamic threshold are selected to guarantee the credibility of task data.

The rest of the paper is organized as follows: related work is reviewed and summarized in Section 2 and Section 3 presents the system model. In Section 4, we quantify user characteristics and analyze their impacts on task completion probability, task data credibility, and ability of participants to complete sensing tasks. In Section 5, participant selection strategies for different heat regions are proposed and the performances of the proposed algorithms are analyzed in Section 6. Finally, we conclude the paper and give some future directions in Section 7.

## 2. Related Work

Many researchers have conducted research on the participant selection and task allocation in MCS. Karaliopoulos et al. propose two greedy heuristic algorithms to recruit some mobile users who can perform location-related sensing tasks with a minimum cost [30]. Zhang et al. study how to select participants to achieve the near-optimal space-time coverage under a limited budget [17]. However, it does not consider the task deadlines and data credibility. Considering the selection of participants with deadlines, especially for time-sensitive sensing tasks, Guo et al. design a greedy-enhanced genetic algorithm that intentionally changes the path and selects participants who can reach the task location with the least moving distance, and design another algorithm for delay-tolerant sensing tasks based on the path coverage history [12]. Although [12] considers the task deadline, it cannot guarantee the data credibility. In addition, a user completes multiple sensing tasks, but the ability of users to complete sensing tasks is not assessed, resulting in a low task completion rate.

To improve the task completion rate, some researchers introduce redundancy to select several participants for the same task [16,31]. Then, methods such as Bayesian inference [32] and majority vote [25] are applied to obtain accurate results. While this solution reduces the impact of incorrect answers on the end result, it increases the budget required to perform a given task. In addition, there is no definition of data quality and the data quality is reported by the user himself/herself, and therefore the data credibility cannot be guaranteed.

Some researchers consider the effect of user characteristics on sensing results in participant selection. In terms of user preferences, Zhang et al. consider that the user has a task preference and allocate tasks that match user preferences as the most valuable sensing tasks to maximize the profits [33]. However, without the users’ willingness to participate and their ability to complete the task accessed, there is uncertainty in the completion time of sensing tasks, and the credibility of the data cannot be guaranteed. Fog computing framework is involved in [24] to evaluate the participant selection factor according to the user’s location, social behavior and the device’s remaining power. The cloud platform selects the participant with the minimum requirements to complete the sensing task. This work considers the user willingness to participate, but it ignores the data credibility issues. Estrada et al. analyze the user reputation, user confidence in completing tasks and the impact of deadlines on data quality. A task allocation architecture based on location and time is proposed to enhance the data quality [29]. However, the proposed architecture ignores the difference in user ability to complete the task and the user willingness to participate, resulting in low task completion rate.

The above studies can achieve their individual optimization goals in high-heat regions. However, these studies can be extended in terms of the wide search range, long waiting time, and low data reliability. In the low-heat regions, the performance of above studies will deteriorate rapidly, resulting in the low task allocation rate and high task failure rate. More importantly, the MCS system is user-centric and users have social characteristics; therefore, the data quality of individual user can be greatly promoted through community assistance. However, these important characteristics are seldom considered by existing research. Therefore, we design a UCPS mechanism for sensing regions of different heat levels to evaluate the completeness of sensing data, data credibility, and task completion time, which can improve the overall data quality and ensure the timely and reliable task completion in low-heat regions.

## 3. System Model

In order to select participants to effectively fulfill sensing tasks and collect sufficient and reliable data, according to the responsibilities, the service platform is divided into three parts: information management module (IMM), participant selection module (PSM) and data service module (DSM). The IMM is responsible for the user registration, real-time status information acquisition, assessment of user willingness, and evaluation and management of user reputation. The PSM is responsible for task publishing and participant selection. The DSM is responsible for receiving service requests, verifying, integrating, and evaluating task data reports.

**Figure 1:**
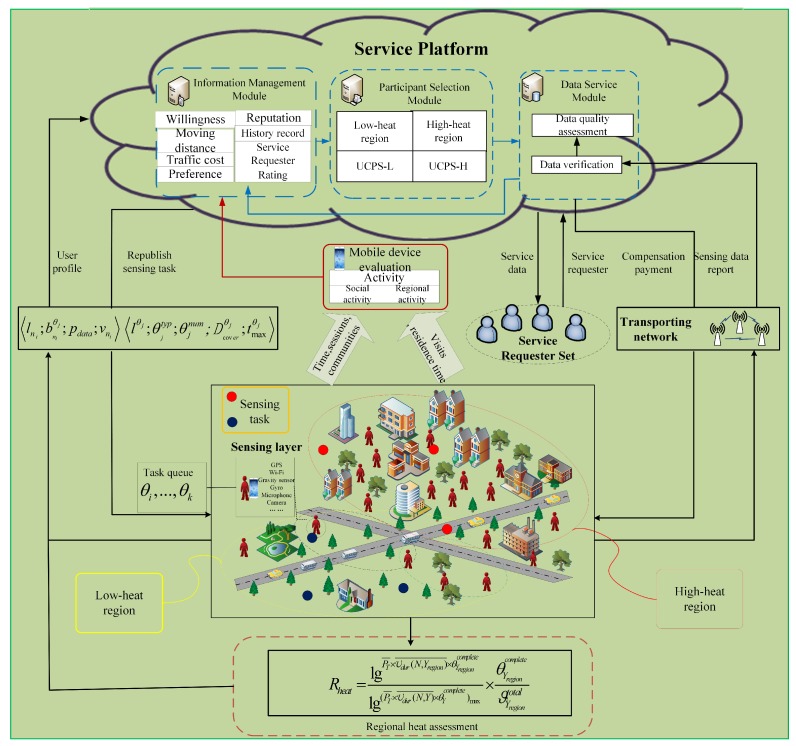
User characteristic aware participant selection process.

The process of MCS participant selection is shown in Figure 1. The MCS system mainly includes service requesters, service platforms and mobile users. The service requester sends the sensing request about certain regions to the service platform, and service requesters are also from users. Each request has a clear type (e.g., photos, videos, and sounds) and deadline. Upon receiving a sensing request, the service platform organizes and classifies the sensing requests, and then publishes a sensing task set $\vartheta =\{\theta_{1},\theta_{2},\ldots ,\theta_{J}\}$, where the budget of each sensing task in the set is $B_{j}$. When a participant completes the task, the payment is $Y_{n_{i}}^{\theta_{j}}$ and cannot exceed the corresponding budget, and therefore profit $\nu_{\theta_{j}}$ gained by service platform is the budget minus the payment. The relevant information of task $\theta_{j}$ is represented by a tuple $\langle l^{\theta_{j}};\theta_{{}_{j}}^{typ};\theta_{j}^{num};D_{{}_{\mathrm{cov}er}}^{\theta_{j}};t_{\max}^{\theta_{j}}\rangle$, indicating the location, task type, required number of participants, coverage radius, and deadline. The set of users in the region is represented as $N=\{n_{1},n_{2},\ldots ,n_{Z}\}$. After receiving the task message, a user submits his/her profile to the service platform and the uploaded file is represented as $\langle {l_{n}}_{{}_{i}},b_{{}_{n_{i}}}^{\theta_{j}},p_{data},v_{n_{i}}\rangle$, which contains the user position, bid, data cost, and speed. IMM evaluates the users’ willingness based on their real-time status information, and PSM selects the set of participants with the highest comprehensive data quality in the high-heat regions $U=\left\{u_{1},u_{2},\ldots ,u_{z}\right\}$. In the low-heat regions, the service platform selects the set of participants who can complete the sensing task before deadline $C=\left\{c_{1},c_{2},\ldots ,c_{y}\right\}$.

## 4. User Characteristics Awareness

The mobility and sociality of mobile users will bring new challenges to MCS services. The success of MCS service depends on not only the service capacity of a provider but also the service environment, including the location information of target regions, user willingness, reputation, and activity. In this section, we first assess the regional heat, further divide the urban space into high-heat and low-heat regions, and then evaluate the user willingness, reputation and activity.

### 4.1. Regional Heat Assessment

The regional heat in this paper indicates the number of users and the probability of completing sensing tasks in a certain region at a given time [29]. Some regions maintain a large number of users and a high probability to complete tasks, whereas other regions do not. According to this obvious difference, urban spaces are divided into high-heat and low-heat regions.

When the sensing tasks are published to a region with a large number of users, the participants can be selected easily and the task can be allocated quickly. However, when the sensing task is published to a region with fewer users, the proper participants in this region are insufficient to complete all the sensing tasks and new arriving users to this region are pending. Therefore, the number of users in a region affects the selection of participants and the convergence of task allocation. Moreover, there are differences in the residence time of users in the region, and the sensing task can only be completed within a sufficient residence time. In addition, a region may have a large number of users, but most of them are not willing to participate in sensing tasks. As a result, the task completion rate is poor. On the contrary, a small number of active users in a given region can complete many sensing tasks and reach a high task completion rate. Hence, the urban spaces are divided into regions with different heat levels, and the regional heat is evaluated according to the number of active users, average residence time of users and history of sensing tasks.

Number of active users $\bar{P_{T}}$ is calculated by the average number of users in region $Y_{region}$ of *T* observations, and $\bar{P_{T}}$ is given by

(1) $$\bar{P_{T}}=\frac{\sum_{i=1}^{T}Total_{t_{i}}^{Y_{region}}}{T},$$

where $Total_{t_{i}}^{Y_{region}}$ is the total number of users in region $Y_{region}$ of the $t_{i}$-th observation and *T* is the total number of observation times.

Next, the average residence time $U_{art}\left(N,Y_{region}\right)$ of all *N* users in $Y_{region}$ can be obtained by

(2) $$\bar{U_{art}\left(N,Y_{region}\right)}=\frac{\sum_{n_{i}\in N}[L\left(n_{i},Y_{region}\right)-ℑ\left(n_{i},Y_{region}\right)]}{N},$$

where $ℑ(n_{i},Y_{region})$ and $L(n_{i},Y_{region})$ are the arrival and departure time of user $n_{i}$.

In this paper, the history of sensing tasks is composed of the number of completed tasks and the task completion rate, and both of them are closely related to the regional heat. In particular, the task completion rate has a significant impact on the platform service performance. Meanwhile, there is a huge difference in the number of active users and completed tasks, and the average residence time of users. In addition, due to the marginal utility, as the values of these three parameters increase, their impact on the regional heat gradually decrease. Therefore, the logarithmic method can be employed to reflect regional heat $H_{region}$. Assuming the total number of sensing tasks completed within the region during *T* observations is $\theta_{Y_{region}}^{{}^{complete}}$ and the total number of tasks published by the service platform for the region is $\vartheta_{Y_{region}}^{total}$, $H_{region}$ can be calculated by

(3) $$H_{region}=\frac{{\mathrm{lg}}^{\bar{P_{T}}\times \bar{U_{dur}\left(N,Y_{region}\right)}\times \theta_{Y_{region}}^{{}^{complete}}}}{{\mathrm{lg}}^{{\left(\bar{P_{T}}\times \bar{U_{dur}\left(N,Y\right)}\times \theta_{Y}^{{}^{complete}}\right)}_{\max}}}\times \frac{\theta_{Y_{region}}^{{}^{complete}}}{\vartheta_{Y_{region}}^{total}},$$

where ${\mathrm{lg}}^{{\left(\bar{P_{T}}\times \bar{U_{dur}\left(N,Y\right)}\times \theta_{Y}^{{}^{complete}}\right)}_{\max}}$ is the maximum value that can be achieved by the product of the average residence time, number of active users and number of completed tasks in all regions.

For the service platform, the proportion of completed sensing tasks and the timeliness of task completion significantly affect the quality of service. Only when the number of completed tasks in a region is less than an expected value can the quality of service of the platform be guaranteed. In all regions that satisfy the number of completed tasks not less than the expected number of tasks, we select the lowest regional heat from these regions compared to the highest regional heat, and use the ratio as the dynamic equilibrium ponit $H_{dep}$

(4) $$H_{dep}=\frac{{\mathrm{lg}}^{{\left(\bar{P_{T}}\times \bar{U_{dur}\left(N,Y\right)}\times \theta_{Y}^{{}^{complete}}\right)}_{\min}}}{{\mathrm{lg}}^{{\left(\bar{P_{T}}\times \bar{U_{dur}\left(N,Y\right)}\times \theta_{Y}^{{}^{complete}}\right)}_{\max}}},\theta_{Y}^{{}^{complete-timely}}\geq \theta_{Y}^{\exp ection},$$

where ${\mathrm{lg}}^{{\left(\bar{P_{T}}\times \bar{U_{dur}\left(N,Y\right)}\times \theta_{Y}^{{}^{complete}}\right)}_{\min}}$ is the minimum value of ${\mathrm{lg}}^{\bar{P_{T}}\times \bar{U_{dur}\left(N,Y\right)}\times \theta_{Y}^{{}^{complete}}}$ when the number of tasks completed in all regions $\theta_{Y}^{{}^{complete-timely}}$ is not less than the expected value $\theta_{Y}^{\exp ection}$. When $H_{region}$ is larger than $H_{dep}$, this region is a high-heat region. When $H_{region}$ is smaller than $H_{dep}$, this region is a low-heat region.

### 4.2. User Willingness

A user’s willingness to participate in a sensing task is usually related to the moving distance, data transmission cost, and preference. Thus, we assess the user willingness as follows.

Tasks published by the service platform are tagged with locations and deadlines and a user arrives at the designated location to complete the sensing tasks within deadlines. The relationship $w_{{}_{dis}}^{n_{i},\theta_{j}}$ between the distance and user willingness to participate can be denoted by

(5) $$w_{{}_{dis}}^{n_{i},\theta_{j}}=\left\{\begin{array}{c} 1,D_{n_{i}}^{\theta_{j}}\leq D_{{}_{\mathrm{cov}er}}^{\theta_{j}}\\ 1-\max\left(\log_{D_{m}}^{\left(D_{n_{i}}^{\theta_{j}}-D_{{}_{\mathrm{cov}er}}^{\theta_{j}}\right)},0\right),D_{{}_{\mathrm{cov}er}}^{\theta_{j}}<D_{n_{i}}^{\theta_{j}}\leq D_{m}\\ 0,D_{n_{i}}^{\theta_{j}}>D_{m}, \end{array}\right.,$$

where $D_{n_{i}}^{\theta_{j}}$ is the Euclidean distance between user location ${l_{n}}_{{}_{i}}$ and task location $l_{\theta_{j}}$, $D_{{}_{\mathrm{cov}er}}^{\theta_{j}}$ is the task coverage radius, and only users within the coverage can participate in the task, and $D_{m}$ represents the maximum acceptable distance.

The service platform is assumed to publish sensing tasks containing images and videos, and users may also upload image and video data to the service platform at high data transmission expense. If a user generates the high expense in traffic, the users’ profits will be reduced even to negative, which also significantly affects the user willingness. Apparently, the higher user profits indicate the higher user willingness to participate. Therefore, the users are divided into a user set $\phi_{1}$ with sufficient traffic and a user set $\phi_{2}$ with restricted data traffic. A user with sufficient data traffic can directly upload data without incurring additional costs, whereas a user with restricted data traffic has to pay for the data uploading. The total cost $C_{\cos t}^{n_{i},\theta_{j}}$ of user $n_{i}$ to complete task $\theta_{j}$ includes data traffic cost $C_{data}$, and power consumption and memory consumption $C_{else}$. According to the profile submitted by user $n_{i}$, the unit traffic cost is $p_{data}$, and the consumed data traffic includes data traffic $o_{\theta_{j}}$ generated by the allocated task and the data traffic of uploading $\int_{0}^{\tau_{trans}}o_{trans}d_{t}$, where $o_{\theta_{j}}$ can be directly reported by the platform, $\int_{0}^{\tau_{trans}}o_{trans}d_{t}$ is closely related to the accuracy of submitted data, and $o_{trans}$ is the data transmission rate. Therefore, the total cost can be given by

(6) $$C_{\cos t}^{n_{i},\theta_{j}}=\left\{\begin{array}{c} c_{else}\;\;\;\;\;\;\;\;\;\;\;\;\;\;\;\;\;\;\;\;\;\;\;\;\;\;\;\;\;\;\;\;\;\;\;\;\;\;\;\;n_{i}\in \phi_{1},\\ p_{data}\left(o_{\theta_{j}}+\int_{0}^{\tau_{trans}}o_{trans}d_{t}\right)+c_{else}\;\;\;\;\;\;\;\;\;\;\;n_{i}\in \phi_{2}. \end{array}\right.$$

We assume that a user can receive payment $Y_{n_{i}}^{\theta_{j}}$ after completing task $\theta_{j}$ and the relationship between the user willingness and total cost $C_{\cos t}^{n_{i},\theta_{j}}$ can be denoted by

(7) $$W_{\cos t}=\max\left[0,\frac{Y_{n_{i}}^{\theta_{j}}-C_{\cos t}^{n_{i},\theta_{j}}}{Y_{n_{i}}^{\theta_{j}}}\right].$$

Obviously, users tend to complete their preferred tasks, thus a high similarity between the types of published tasks and the preferred tasks signifies a high user inclination to participate. When a user registers for the first time, the MCS system allows the user to rate task types according to the preference, and then the project-based collaborative filtering is employed to recommend sensing tasks for the user. The interested sensing tasks should be the highly rated task types. Hence, the key of collaborative task type filtering lies in the similarity between tasks [34]. Mutual information can measure the similarity between two subsets of data from the same data set. Each task type contains the class identifier and belongs to the cluster. Therefore, the mutual information idea is employed to measure the similarity between two clusters. We use $\sigma_{1}$ as the user preference for task types, and $\sigma_{2}$ as the sensing tasks published by the service platform, and $\upsilon_{\iota },\iota \in \left\{1,\ldots ,\delta \right\}$ and $\varpi_{h},h\in \left\{1,\ldots ,\phi \right\}$ as the class identifiers for $\sigma_{1}$ and $\sigma_{2}$, respectively, and then the similarity between $\sigma_{1}$ and $\sigma_{2}$ is given by

(8) $$I\left(\sigma_{1},\sigma_{2}\right)=\sum_{\upsilon_{\iota }\in \sigma_{1}}\sum_{\varpi_{h}\in \sigma_{2}}p(\upsilon_{\iota },\varpi_{h})\log\frac{p(\upsilon_{\iota },\varpi_{h})}{p\left(\upsilon_{\iota }\right)p\left(\varpi_{h}\right)},$$

where $p\left(\upsilon_{\iota }\right)=\left|\upsilon_{\iota }\right|/n,p\left(\varpi_{h}\right)=\left|\varpi_{h}\right|/n,p\left(\upsilon_{\iota },\varpi_{h}\right)=\left|\upsilon_{\iota }\cap \varpi_{h}\right|/n$, *n* is the number of samples.

In fact, mutual information is biased toward variables with multiple values and the standardized mutual information can correct this bias with a value range in $\left[0,1\right]$. By combining information entropies of $\sigma_{1}$ and $\sigma_{2}$ with their similarity, we can standardize the mutual information. The information entropy can reflect the probability of certain task types appearance, and the standardized form of mutual information can be given by

(9) $$\Omega \left(\sigma_{1},\sigma_{2}\right)=\frac{I(\sigma_{1},\sigma_{2})}{\sqrt{H\left(\sigma_{1}\right)*H\left(\sigma_{2}\right)}},$$

where $H(\sigma_{1})$ and $H(\sigma_{2})$ denote the information entropies of $\sigma_{1}$ and $\sigma_{2}$, respectively.

The similarity between $\sigma_{1}$ and $\sigma_{2}$ reflects the user willingness to participate in sensing tasks, namely $W_{preference}=\Omega \left(\sigma_{1},\sigma_{2}\right)$.

In summary, the moving distance, data transmission cost, and task type preference affect the user willingness. Users with higher willingness are more likely to participate in sensing tasks, and thus to accomplish tasks. The data transmission cost inevitably affects the user willingness and the higher payment also incurs the higher willingness. Even if the task type is not preferred, the user will still participate in sensing tasks due to the incentive payments. Eventually, the user willingness can be denoted by

(10) $$W_{n_{i}}=w_{{}_{dis}}^{n_{i},\theta_{j}}\times \frac{1+w_{preference}}{2}\times \left(e^{w_{\cos t}}-1\right).$$

### 4.3. User Reputation

Reputation is an important parameter to measure the credibility of sensing data [35]. However, the existing MCS research reputation evaluates the user reputation only according to the task participation records and ignores the experience of service requester, which is considered in this paper as a metric to assess the provided service according to the actual experience and satisfaction. Therefore, the user reputation should be comprehensively evaluated based on the scores from service requesters and the task participation records.

In detail, the time span of user $n_{i}$ participating in the sensing task is counted by days and denoted by $T_{day}$. When updating the cumulative scores, we should give more weight to the more recent records. In addition, with the growing participation time, the participation records of the user also increase accordingly. The more scoring stages lead to the more accurate cumulative scores. We use the logarithm of Euler’s number $\left\lceil \ln^{T_{day}+\ell }\right\rceil$ to divide the participation records into $T_{{}_{stage}}^{\max}$ time periods, which can update user reputation according to the impact of the recent and old records. Due to the exponential attenuation, the participation records before five time periods have less weight. Thus, the cumulative score of users can be denoted by

(11) $$\delta_{n_{i}}=\sum_{T_{stage}=1}^{\min(T_{stage}^{\max},5)}\sum_{\chi =1}^{Q}{\left(\frac{1}{2}\right)}^{T_{stage}}\bar{\delta_{n_{i},score}^{T_{stage},\chi }},$$

where *Q* represents the number of tasks completed by user $n_{i}$ in $T_{stage}$ stages and $\bar{\delta_{n_{i},score}^{T_{stage},\chi }}$ represents the average score of $n_{i}$ in stage $T_{stage}$. The cumulative score is then compared with the average of all registered users in the service platform. Furthermore, ratio α can be used as the reputation overlay coefficient to more accurately measure the user reputation, α as defined by

(12) $$\alpha =\frac{\delta_{n_{i}}}{\sum_{i=1}^{Z}\delta_{n_{i}}/Z}.$$

The larger value of α signifies the higher reliability. If a user has completed all tasks assigned by the platform but his/her ratio is relatively low, the user probably submits some invalid data and the ratio serves as the penalty factor to reduce the user reputation.

The participation records of users include the completed tasks acknowledged by the platform, completed tasks with invalid data, and uncompleted tasks. To prevent users from repeatedly submitting task data to improve their reputation in a short period of time for the later malicious attempts, the increase of user reputation should be insensitive to the consecutive submissions of task data. When a user submits invalid data, his/her reputation should be significantly reduced. The ratio of completed tasks with invalid data acknowledged by the platform serves as the coefficient to attenuate the user reputation. For uncompleted tasks, the service platform slows down the decline in reputation. Combining the participation records of user $n_{i}$ and reputation overlay factor α, we can obtain the user reputation of $n_{i}$ by

(13) $${ℜ}_{n_{i}}^{reputetion}=\min\left\{1,\alpha \frac{\vartheta_{n_{i}}^{True}}{\vartheta_{n_{i}}^{Total}\times e^{\frac{\vartheta_{n_{i}}^{Invalid}}{\vartheta_{n_{i}}^{{}_{Complete}}}}}\right\},$$

where $\vartheta_{n_{i}}^{{}_{Total}}$ is the total number of tasks accepted by user $n_{i}$, $\vartheta_{n_{i}}^{True}$ is the number of completed tasks acknowledged by the platform, $\vartheta_{n_{i}}^{Complete}$ is the number of tasks completed by user $n_{i}$, and $\vartheta_{n_{i}}^{Invalid}$ is the number of completed tasks with invalid data.

### 4.4. User Activity

We notice that the home communities of users include not only traditional offline communities but also various online communities. Obviously, users may belong to multiple communities and users with high regional activity are more likely to visit more locations and thus able to accomplish more sensing tasks. Moreover, users interact with others from different communities and form a sensing network covering the real-world communities. The users with a high amount of social activity have a wide social sensing network, which are stable and reliable, and can help the user complete more sensing tasks. Therefore, the regional activity and the social activity are combined to obtain the user activity.

#### 4.4.1. Regional Activity

According to the observations of users’ trajectories and behaviors, user movements are event-driven [36]. The visiting times to region $m_{k}$ and the average residence time can reflect the user activity in region $m_{k}$. The more visits signify the greater probability of arriving at the region, the longer average residence time and the enhanced possibility of completing the sensing task.

The probability $P(n_{i},m_{k})$ of user $n_{i}$ visiting region $m_{k}$ can be obtained by comparing the visiting times $z_{num}\left(n_{i},m_{k}\right)$ to region $m_{k}$ with the total visiting times to all regions $\sum_{m_{j}\in M}z_{num}\left(n_{i},m_{j}\right)$, as shown as follows:

(14) $$P\left(n_{i},m_{k}\right)=\frac{z_{num}\left(n_{i},m_{k}\right)}{\sum_{m_{j}\in M}z_{num}\left(n_{i},m_{j}\right)}.$$

The residence time of user $n_{i}$ at $m_{k}$ can be expressed by the difference between departure time $e(n_{i},m_{k})$ and arrival time $s(n_{i},m_{k})$ and the average residence time of user $n_{i}$ at $m_{k}$ in *K* visits can be denoted by

(15) $$H_{dur}\left(n_{i},m_{k}\right)=\frac{\sum_{m_{k}\in M}\left[e\left(n_{i},m_{k}\right)-s\left(n_{i},m_{k}\right)\right]}{T}.$$

Specifically, both the visiting probability and the average residence time are highly related to the users’ activity in a region. Therefore, the product of the visiting probability and the average residence time in $m_{k}$ can be exploited as the regional activity factor, i.e.,

(16) $$R_{region}^{n_{i}}=P\left(n_{i},m_{k}\right)\times H_{dur}\left(n_{i},m_{k}\right).$$

In order to intuitively reflect the regional activity of users and properly set its value range, we map $R_{region}^{n_{i}}$ with the Min-Max normalization into [0,1]. Then, the regional activity can be obtained by

(17) $$R_{n_{i}}=\frac{R_{region}^{n_{i}}-R_{N}^{\min}}{R_{N}^{\max}-R_{N}^{\min}},$$

where $R_{N}^{\max}$ and $R_{N}^{\min}$ are the maximum and minimum regional activity among *N* users.

#### 4.4.2. Social Activity

Users interact with the service platform when participating in sensing tasks and the service platform expects users to rapidly respond to and actively participate in sensing tasks. In daily life, some users use their devices frequently and belong to multiple online communities, making them ideal task participants, and we regard these users as high social activity users. User interactions in social applications (e.g., Weibo, Facebook and Twitter) can be depicted by the session duration, data usage and location detection information [37]. The social activity involved in this paper is determined by the time spent on social applications, the number of established sessions during the time and the number of online communities connected by these sessions.

Generally speaking, user behavior changes as the social environment changes. Certain long-term user behaviors can be maintained, and changes in social behaviors are triggered by recent short-term events. Therefore, the social activity can be divided into the short-term and long-term social activity according to user behaviors. The short-term social activity $S_{{}_{n_{i}}}^{short}$ of user $n_{i}$ is closely related to the change in social environment. In particular, when there is a significant change in the number of users interacting with user $n_{i}$ and the number of communities in which user $n_{i}$ is active, we adopt the relatively stable short-term social activity. Due to the event-driven impact, short-term changes in users and online communities will significantly affect the social activity. After multiplying the user number change by the community number change, we compare the result with the number of users with sessions established or the number of active online communities. If the obtained product is greater than any of them, it indicates a significant change in user status. Then, the time period before and after the change can be exploited to statistically evaluate the short-term activity, and the latest period is denoted by $T_{1}$, the longest period is denoted by $T_{\omega }$ short-term social activity $S_{{}_{n_{i}}}^{T_{i}-short}$ of user $n_{i}$ in period $T_{i}$ can be obtained by

(18) $$S_{{}_{n_{i}}}^{T_{i}-short}=\frac{\left(\sum t_{i}^{u_{a_{x}}^{u}}\right)}{T_{i}}\times s_{n_{i}}^{T_{i}-sessions}\times s_{n_{i}}^{\bar{T_{i}-SNGroup}},$$

where $\sum t_{i}^{u_{a_{x}}^{u}}$ is the total time spent on social applications in period $T_{i}$, $s_{n_{i}}^{T_{i}-sessions}$ is total amount of sessions of user $n_{i}$ in time period $T_{i}$, and $s_{n_{i}}^{\bar{T_{i}-SNGroup}}$ is the number of communities within period $T_{i}$ with sessions established by user $n_{i}$ exceeding the average number of sessions of all his online communities. After obtaining the short-term activity within each period, we further analyze the correlation between the short-term and long-term activity. In fact, the longer update cycle signifies the more stable short-term activity and the more accurate long-term activity. In addition, the update cycle has a great impact on the long-term activity. On the contrary, the more frequently updated short-term activity signifies the more unstable user activity, and less weight on the long-term user activity. Thus, long-term user activity $S_{{}_{n_{i}}}^{long}$ can be calculated by

(19) $$S_{{}_{n_{i}}}^{long}=\sum_{i=1}^{\omega }\frac{T_{i}}{\sum_{i=1}^{\omega }T_{i}}S_{{}_{n_{i}}}^{T_{i}-short}.$$

The logarithmic function does not change the nature and correlation of the data, but it compresses the variable scale, facilitates calculation, and intuitively reflects the social activity. Thus, $S_{{}_{n_{i}}}^{all}$ is mapped into [0,1] using Min-Max normalization and logarithmic function, to obtain social activity $S_{n_{i}}$,

(20) $$S_{n_{i}}=\frac{\log S_{{}_{n_{i}}}^{all}-\log S_{N}^{\min}}{\log S_{N}^{\max}-\log S_{N}^{\min}},$$

where $S_{N}^{\max}$ and $S_{N}^{\min}$ are the maximum and minimum long-term social activity among *N* users, respectively.

Analytically, the user activity includes regional activity and social activity. The influence of regional activity and social activity on sensing tasks in regions with different heat levels vary largely. Therefore, the regional activity and social activity should be analyzed comprehensively, as denoted by

(21) $$A_{n_{i}}=\gamma S_{n_{i}}+\left(1-\gamma \right)R_{n_{i}}.$$

Obviously, the weight factor is crucial to the measurement of user activity. There are more potential participants in high-heat regions, and users with higher activity can reduce the interaction time with the service platform. Therefore, the weight factor should be adjusted according to the regional heat. In low-heat regions, sensing tasks have to be accomplished in a timely and reliable manner and the strong task completing ability of users with high regional activity should be fully considered. Therefore, weight factor γ in this paper is assumed to be equal to regional heat $H_{region}$.

## 5. Participant Selection Strategy

Participants can accept multiple sensing tasks and selectively perform tasks with short deadlines. For tasks with long deadlines, participants store them in terminal devices and complete them according to their priorities. The shorter deadline signifies the higher sensing task priority. Specifically, it is difficult for platforms to select suitable participants from low-heat regions and selected participants may also fail in completing the assigned tasks due to unexpected events. However, users form online communities in a self-organizing manner and users in the same communities have stable and relatively close social relationships. The social relationship of MCS systems can be exploited to expand the sensing coverage. Therefore, the task queuing strategy and community assistance strategy are introduced to ensure the efficient and reliable task performance [38,39]. Furthermore, the service platform combines user characteristics and bid evaluation to enhance the data quality, and then selects the user with the highest data quality as the participant in high-heat regions. In low-heat regions, the service platform selects participants with profit exceeding the dynamic threshold.

### 5.1. Task Queueing Strategy and Community Assistance Strategy

Participants employ the task queuing strategy to complete the accepted tasks in order. When designing the task queuing strategy, we consider that users with higher activity in the same network community have close connection with other users. When users encounter an inevitable event interrupting the task completion, they may recommend other users to assist in completing sensing tasks, thereby ensuring the task completion rate. Correspondingly, the number of task queues is highly related to the user activity. In addition, the service platform should set the upper and lower limits based on the number of queued tasks to ensure the quality of service. For the case that users assist in completing tasks, sensing tasks should be completed under the remaining budget and time constraints, while ensuring the data quality.

#### 5.1.1. Task Queueing Strategy

When a user participates in tasks, the service platform will set the minimum and maximum number of tasks to be completed. In addition, due to the different ability, the number of tasks assigned for each user varies. Therefore, the number of tasks accepted by users should be restricted accordingly. The maximum number of consecutive tasks stored by user $n_{i}$ is denoted by $Q_{n_{i}}^{\max}$. According to Section 4.4, users with high activity are likely to complete more sensing tasks, and $Q_{n_{i}}^{\max}$ should change linearly with user activity $A_{n_{i}}$. Therefore, $Q_{n_{i}}^{\max}$ can be represented by the minimum number of tasks a user needs to complete plus the number of tasks completed due to user activity. In addition, in order to reliably complete sensing tasks, the upper limit of sensing tasks that each user can accept is set to $Q^{\max}$ and $Q_{n_{i}}^{\max}$ cannot exceed $Q^{\max}$. Thus, $Q_{n_{i}}^{\max}$, i.e.,

(22) $$Q_{n_{i}}^{\max}=\left\lfloor Q_{n_{i}}^{base}+A_{n_{i}}\left(Q^{\max}-Q_{n_{i}}^{base}\right)\right\rfloor .$$

Obviously, when a user participates in tasks, his willingness will affect the number of accomplished sensing tasks. Therefore, the service platform dynamically adjusts the upper limit of the queue according to the user willingness and the number of queued sensing tasks cannot exceed the adjusted upper limit $Q_{n_{i}}^{r-\max}$, i.e.,

(23) $$Q_{n_{i}}^{r-\max}=W_{n_{i}}\times Q_{n_{i}}^{\max}.$$

Furthermore, we assume that the number of sensing tasks that user $n_{i}$ actually accepts is $Q_{n_{i}}^{fact}$ and the accepted sensing tasks are stored in the mobile device according to the deadlines and denoted by set $\vartheta {}_{n_{i}}^{\prime }$. The payment $Y_{n_{i}}^{total}$ for completing tasks $\theta_{j}$ cannot exceed total budget $\sum_{j=1}^{Q_{n_{i}}^{fact}}B_{j}$. Assume that there are $Q_{n_{i}}^{fact}$ sensing tasks in $\vartheta {}_{n_{i}}^{\prime }$ and the payment for completing task $\theta_{j}$ is $Y_{n_{i}}^{\theta_{j}}$, and then the relationship between payment and budget can be denoted by

(24) $$Y_{n_{i}}^{total}=\sum_{j=1}^{Q_{n_{i}}^{fact}}Y_{n_{i}}^{\theta_{j}}\leq \sum_{j=1}^{Q_{n_{i}}^{fact}}B_{j}.$$

Since the user uploads the sensing data to the platform after completing a task in the queue, the total time that user $n_{i}$ completes the tasks in the queue cannot exceed the longest deadline, satisfying

(25) $$\frac{D_{n_{i}}^{\theta_{j}}+\sum_{j=1}^{Q_{n_{i}}^{fact}}dis_{\theta_{j}}}{v_{n_{i}}}+\sum_{j=1}^{Q_{n_{i}}^{fact}}t_{n_{i}}^{\theta_{j}}\leq T_{\theta_{j}}^{\max},$$

where $\sum_{j=1}^{Q_{n_{i}}^{fact}}dis_{\theta_{j}}$ is the total moving distance to complete the queued tasks, $\sum_{j=1}^{Q_{n_{i}}^{fact}}t_{n_{i}}^{\theta_{j}}$ is the total time of completing the queued tasks, and $T_{\theta_{j}}^{\max}$ denotes the longest deadline for all accepted tasks.

Similarly, the service platform selects participants according to the task priority. If no participant is performing a sensing task, the sensing task is queued until any participant is available. The shorter task deadline signifies the higher task priority. If any queued task expires, it is removed from the queue and marked as an uncompleted task.

#### 5.1.2. Community Assistance Strategy

The community assistance strategy is designed in this paper to screen participants who are unable to complete their assigned tasks due to unpredictable circumstances. We consider that participant $u_{i}$ who is unable to complete the tasks can recommend one or a set of users from the social network to complete his assigned tasks. Since the MCS system may not know the situation, the PSM selects a set of participants $N_{u_{i}}^{appropriate}$, who are registered in the system from and can meet the following requirements from the recommended user set.

The maximum budget to be allocated to $N_{u_{i}}^{appropriate}$ should be less or equal to the remaining budget.

(26) $$Y_{N_{u_{i}}^{appropriate}}\leq \sum_{j=1}^{Q_{u_{i}}^{fact}}B_{j}-\sum_{j=1}^{Q_{nu}^{fact}}Y_{u_{i}}^{\theta_{j}}.$$

When community assistance is enabled, $N_{u_{i}}^{appropriate}$ may be located outside of the task coverage area. However, they should reach the task location within the remaining response time, as restricted by

(27) $$t_{N_{u_{i}}^{appropriate}}^{\theta_{j}}\leq T_{\theta_{j}}^{\max}-\left(\frac{D_{u_{i}}^{\theta_{j}}+\sum_{j=1}^{Q_{u_{i}}^{fact}}dis_{\theta_{j}}}{v_{u_{i}}}+\sum_{j=1}^{Q_{u_{i}}^{fact}}t_{u_{i}}^{\theta_{j}}\right).$$

The data quality that $N_{u_{i}}^{appropriate}$ can achieve should not be lower than that of $u_{i}$, and the data quality evaluation is crucial. The probability of not completing tasks by $N_{u_{i}}^{appropriate}$ is exponentially reduced, and therefore only one assistance is taken into account, which means that $N_{u_{i}}^{appropriate}$ are not all allowed to assist. Thereby, $u_{i}$ can avoid the bad reputation for future tasks.

### 5.2. Participant Selection Strategy for High-Heat Regions

We employ the method proposed in Section 4.1 to divide the urban spaces into sensing regions with different heat levels. The service platform publishes sensing tasks in high-heat regions and there are multiple users available to participate. However, the difference of users’ willingness incurs various task completing probabilities. Moreover, the user reputation also varies and results in the different data credibility. Therefore, the service platform employs the multi-attribute reverse auction method [40,41,42] to select participants with the highest comprehensive data quality for different sensing tasks [43].

When the service platform selects participants for task $\theta_{j}$, it firstly collects the submitted user profiles and evaluates the user reputation and activity through methods proposed in Section 4.3 and Section 4.4, respectively. Subsequently, the comprehensive data quality is calculated according to the bid, and the service platform allocates sensing tasks to selected participants and receives the data uploaded by the participants, so as to ensure the data integrity. By selecting participants with high reputation, the data accuracy can be guaranteed. Moreover, the selected participants can reduce the overall payment, gain more profit, consume less time in task completion and achieve better data timeliness and higher data reliability. Data quality $QoC_{{}_{n_{i}}}^{\theta_{j}}$ obtained by user $n_{i}$ can be calculated by

(28) $$QoC_{{}_{n_{i}}}^{\theta_{j}}=\frac{{ℜ}_{n_{i}}^{reputetion}}{Y_{n_{i}}^{\theta_{j}}\times t_{-total}},$$

where $Y_{n_{i}}^{\theta_{j}}$ is the payment for user $n_{i}$ completing task $\theta_{j}$, the payment is equal to bid $b_{n_{i}}^{\theta_{j}}$, and $t_{-total}$ is the total interaction time between user $n_{i}$ and the service platform for data uploading, which can be calculated by

(29) $$t_{-total}=D_{n_{i}}^{\theta_{j}}/v_{n_{i}}+\bar{t_{-interactive}}\times A_{n_{i}}+t_{-collect},$$

where $\bar{t_{-interactive}}$ is the average interaction time between the user and the platform and $t_{-collect}$ is the completion time of task $\theta_{j}$.

According to the analysis in Section 4.2, the user willingness reflects the task completing probability and the expected data quality of user $n_{i}$ can be obtained by

(30) $$\bar{QoC_{{}_{n_{i}}}^{\theta_{j}}}=w_{n_{i}}^{\theta_{j}}\times \frac{{ℜ}_{n_{i}}^{reputetion}}{Y_{n_{i}}^{\theta_{j}}\times t_{-total}}.$$

In high-heat regions, user $n_{i}$ is considered to only participate in the auction of a single sensing task at a time, so as to improve the data quality. If task $\theta_{j}$ is allocated to user $n_{i}$ who is participating in task $\theta_{k}$, the available time of user $n_{i}$ changes and the time to complete task $\theta_{k}$ increases to the time of completing the two tasks. The comprehensive data quality of task $\theta_{k}$ by user $n_{i}$ can be obtained by

(31) $$\bar{QoC_{{}_{n_{i}}}^{\theta_{k}}}=w_{n_{i}}^{\theta_{k}}\times \frac{{ℜ}_{n_{i}}^{reputetion}}{Y_{n_{i}}^{\theta_{k}}\times \sum_{j=1}^{Q_{n_{i}}^{fact}}t_{total}}.$$

When user $n_{i}$ is assigned no less than two sensing tasks, these tasks are completed in accordance with the queuing strategy. The service platform evaluates the comprehensive data quality of sensing tasks, and then selects participants according to the evaluation results. Particularly, the participant selection problem can be transformed into finding a subset *U* in set *N*, which maximizes the overall data quality when the sensing tasks in set ϑ are covered by set *U*. Furthermore, this problem was proved to be the classic NP-hard set coverage problem [12]. For subset *U* in given set *N*, we can define a utility function $F_{U}\left(\vartheta \right)$ of *U* to show how much the selected participants in *U* can achieve data quality. $F_{U}\left(\vartheta \right)$ is non-negative, monotonic and submodular, and the corresponding proofs are given in Appendix A. The maximum data quality is highly related to the budget and deadline. According to the nature of submodule functions, for any U⊆N, $F_{U}\left(\vartheta \right)>0$. A greedy algorithm can find the near-optimal solution [10]. Therefore, the service platform can exploit the greedy algorithm to iteratively search and select participants. In each iteration, the user maximizing utility function $F_{U}\left(\vartheta \right)$ is selected. Specifically, selected participant $n_{i}$ during the *i*-th iteration satisfies the condition in

(32) $$F_{U_{i}}\left(\vartheta \right)=F_{U_{i-1}}\left(\vartheta \right)\cup \left\{\arg\max\Delta F_{(n_{i}\left|U_{i-1})\right.}\left(\vartheta \right)\right\},$$

where $\Delta F_{(n_{i}\left|U_{i-1})\right.}\left(\vartheta \right)=F_{\left(U_{i-1}\cup \left\{n_{i}\right\}\right)}\left(\vartheta \right)-F_{U_{i-1}}\left(\vartheta \right)$. When the service platform allocates all sensing tasks or the budget runs out, the participant selection process terminates, as shown in Algorithm 1.

**Algorithm 1** User Characteristic Aware Participant Selection for High-Heat Regions (UCPS-H)**Input:** Task set ϑ, User set *N*; **Output:** 1: Participant $u_{i}$ selected for task $\theta_{j}$, the payment for participants $Y_{c_{i}}^{total}$ and participants set *U*; 2: $n_{i}\leftarrow 1\;to\;\left|Z\right|,\theta_{j}\leftarrow 1\;to\;\left|\vartheta \right|$; 3: **while**
$bid_{n_{i}}^{\theta_{j}}\leq B_{\max}^{\theta_{j}}$
**do** 4:  Calculate the comprehensive data quality of users for task $\theta_{j}$ through Equation (31); **and** rank the comprehensive data quality of users in descending order 5:  Assign task $\theta_{j}$ to user $n_{i}$ with the highest comprehensive data quality; 6:  $U\leftarrow \arg\max\bar{QoC_{n_{i}}^{\theta_{j}}}$; 7:  $Y_{n_{i}}^{\theta_{j}}\leftarrow bid_{n_{i}}^{\theta_{j}}$; 8:  $\theta_{j}\leftarrow \theta_{j+1}$; 9:  **return** to 3; 10:  **if** task $\theta_{j}$ achieves the desired data quality **then** 11:    remove it from ϑ; 12:  **end if** 13:  **if** all tasks are assigned or budget runs out **then** 14:    stop the selection process; 15:  **end if** 16: **end while** 17: **return**
$\langle X_{n_{i}}^{\theta_{j}},Y_{n_{i}}^{\theta_{j}},U\rangle$;

### 5.3. Participant Selection Strategy for Low-Heat Regions

When the service platform collects data from low-heat regions, there are fewer potential participants, but most users in the low-heat regions have longer residence time and more locations to visit, so the users who can complete multiple sensing tasks should be selected as participants. In low-heat regions, the service platform faces challenges because users arrive at low-heat regions in the random order must complete a certain number of tasks before the deadline, and have to upload the credible sensing data. The multi-stage sampling-reception method can dynamically expand the number of user sets and derive a threshold for each stage based on the user characteristic and platform budget. After the selection of participants at each stage, the thresholds are dynamically updated to solve these three challenges. Specifically, we divide the participant selection process into $1,2,\ldots ,\left|\log_{2}^{T}\right|,\left|\log_{2}^{T}\right|+1$ stages, where stage *k* ends in $T_{k}=\left\lfloor 2^{k-1}T/2^{\left[\log_{2}^{T}\right]}\right\rfloor$, and the budget of stage *k* is $B^{k}=\left\lfloor 2^{k-1}B/2^{\log_{2}^{T}}\right\rfloor$. In the initial stage, the observed profits come from users submitting profiles, and the threshold is set according to the average profits to select participants in the subsequent stages.

According to Section 5.1, the proposed task queuing strategy and community assistance strategy can motivate users with high activity to increase the number and probability of completing tasks and ensure data credibility. When the number of tasks selected by the user does not exceed the number of remaining tasks in this stage, the number of tasks actually accepted by the user is $Q_{n_{i}}^{fact}$. After completing the sensing task, a user will produce certain profit for the platform, and data of higher credibility and integrity bring more profit. Thus, the total profit of user $n_{i}$ can be obtained by

(33) $$\varsigma_{{}_{total}}^{n_{i}}={ℜ}_{n_{i}}^{reputetion}\times \sum_{j=1}^{Q_{n_{i}}^{fact}}\left(\theta_{j}\times \nu_{\theta_{j}}\right).$$

In this paper, the threshold is based on profiles submitted by *k* users during the initial stage, as denoted by

(34) $$\rho =\frac{\sum_{i=1}^{\kappa }(\varsigma_{{}_{total}}^{n_{i}})}{\kappa }.$$

The service platform dynamically adjusts the number of users and budget. When user $n_{i}$ submits his/her profile at time *t*, the service platform checks the profit of users and then selects participants. After the participant selection, the threshold is updated based on the average profit of all selected participants at the given stage. Therefore, the service platform can collect sufficient and reliable sensing data from low-heat regions, and reduce the total number of selected participants and the platform overhead. The specific participant selection process is shown in Algorithm 2.

**Algorithm 2** User Characteristic Aware Participant Selection for Low-Heat Regions (UCPS-L)**Input:** 1: Task set ϑ, Budget *B*, Deadline *T*; **Output:** 2: The task set $\vartheta {}_{c_{i}}$ allocated to participant $c_{i}$, the payment $Y_{c_{i}}^{total}$ and participant set *C*; 3: $\left(t,T^{\prime },B^{\prime },\rho ,C,N\right)\leftarrow \left(1,T/2^{\left\lfloor \log_{2}^{T}\right\rfloor },B/2^{\left\lfloor \log_{2}^{T}\right\rfloor },\ell ,\emptyset ,\emptyset \right)$; 4: **if**
t≤T
**then** 5:  add user arriving at t to online active user set *N*; 6:  $N\leftarrow N\cup \{n_{i}\}$; 7: **end if** 8: **while**
(24)&&(25)
**do** 9:  Compute threshold according (34); 10:  **if**
$\varsigma_{{}_{total}}^{n_{i}}>\rho$
**then** 11:    $\vartheta_{c_{i}}\leftarrow \left\{\theta {}_{1}^{\prime },\theta {}_{2}^{\prime },\ldots ,\theta {}_{Q_{c_{i}}^{fact}}^{\prime }\right\};Y_{c_{i}}^{total}\leftarrow \sum_{j=1}^{Q_{c_{i}}^{fact}}Y_{c_{i}}^{\theta {}_{j}^{\prime }};C\leftarrow C\cup \left\{u_{i}\right\})$; 12:  **end if** 13:  **if** there are still remaining tasks in stage $\log_{2}^{T}$
**then** 14:    **return** to 10; 15:  **else** 16:    all tasks in stage $\log_{2}^{T}$ are allocated, remove the allocated tasks; 17:  **end if** 18:  according (34) update threshold ρ; 19: **end while** 20: **if**
$t=\left\lfloor T^{\prime }\right\rfloor$
**then** 21:  $T^{\prime }\leftarrow 2T^{\prime };B^{\prime }\leftarrow 2B^{\prime }$; 22:  t←t+1; 23: **end if** 24: **return**
$\langle \vartheta {}_{c_{i}},Y_{c_{i}}^{total},C\rangle$;

## 6. Numerical Results

In this paper, we employ datasets derived from Brightkite (Los Angeles, CA, USA), a location-based social network service provider. Users can share their locations by signing in and the service provider can collect user information through public API and form an undirected friendship network, which consists of 58,228 users and 214,078 sides. The user information includes user number, access time, latitude, longitude, and check-in location ID. The 100-location subset and 1224-user subset are extracted to represent task locations and participants in an actual MCS scenario. The main simulation parameters are given in Table 1.

To evaluate the performance, we add some constraints to benchmark algorithms including secure user recruitment (SUR) [44] and greedy-enhanced genetic algorithm for intentional movement (GGA-I) [12]. The SUR algorithm selects the user with the highest data quality, whereas the GGA-I preferentially selects the user with the shortest moving distance to perform sensing tasks.

### 6.1. The Impact of User Characteristics on Task Allocation Rate and Task Completion Ratio

**Figure 2:**
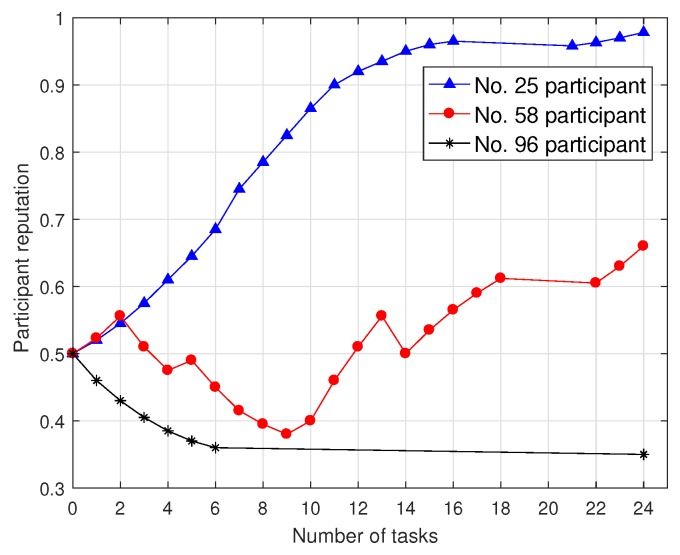
Participant reputation under a various number of tasks.

To evaluate the users’ behaviors and their impacts on reputation, we divide participants into always positively sensing, always negatively sensing and intermittently negative sensing participants, and give participants No. 25, No. 58 and No. 96 for instance. The initial reputation of participants is set to 0.5. As shown in Figure 2, the participant reputation changes along with the participation times and the number of completed tasks. Participant No. 25 is always positively sensing from task 0 to 16 and the reputation rises smoothly from 0.5 to 0.96. Since participant No. 25 does not participate in tasks 16 to 21, his/her reputation is attenuated to 0.95. As participant No. 25 continues to participate in task 22 to 24, his/her reputation slowly increases. Since participant No. 96 has six consecutive negative sensing, his/her reputation declines sharply from 0.5 to 0.35, and participant No. 96 is never selected as a participant again. Participant No. 58 is intermittently negative sensing, and his/her reputation fluctuates with his sensing behaviors. However, with the increasing number of invalid data submitted by the participant, the service platform gradually heightens the penalty. Apparently, the decline in participant reputation results in the gradually increased reputation recovery period because the reputation update mechanism proposed in this paper punishes negative sensing behaviors. The more negative sensing behaviors signify the faster reputation drop. In addition, the attenuation factor is introduced and the participant reputation attenuates over time.

**Figure 3:**
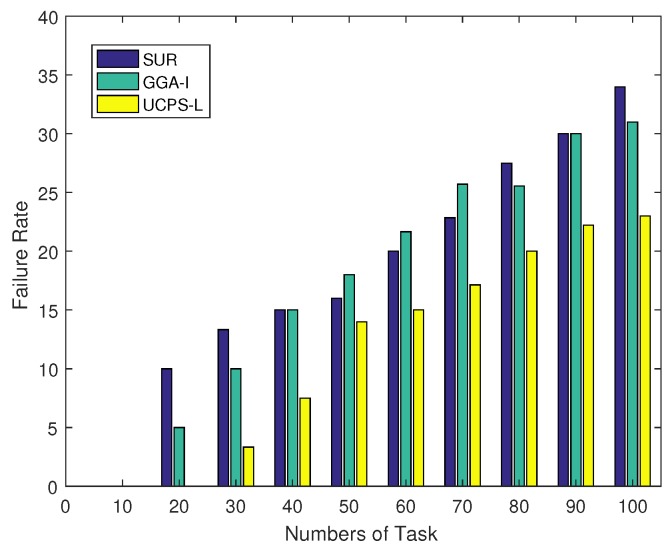
Failure rate under a various total number of tasks.

In terms of the relationship between the user characteristics and task failure rate, Figure 3 illustrates the task failure rates of the three algorithms as the total number of tasks increases in low-heat regions. The task failure rate in this paper is derived from the number of uncompleted tasks, the number of unallocated tasks and the total number of tasks. When the total number of tasks is less than 80, the task failure rate of GGA-I is always higher than those of SUR and UCPS-L. When the total number of tasks reaches 90, the performance of SUR deteriorates sharply and its task failure rate exceeds 16.7% because GGA-I exploits only a single participant to complete multiple sensing tasks. When the number of tasks is small and the sensing region is wide, the moving distance of users is long and the task failure rate increases. However, when the service platform publishes more than 80 tasks, SUR spends more budgets to select high-quality participants in the early stage, resulting in some unallocated tasks and the rapidly increased failure rate. On the contrary, the proposed UCPS-L analyzes the user ability to complete tasks and selects reliable participants based on the user reputation to ensure the completion of tasks. When the total number of allocated tasks by the service platform reaches 100, the failure rate of UCPS-L is 66.7% and 50.6% lower than those of SUR and GGA-I, respectively.

**Figure 4:**
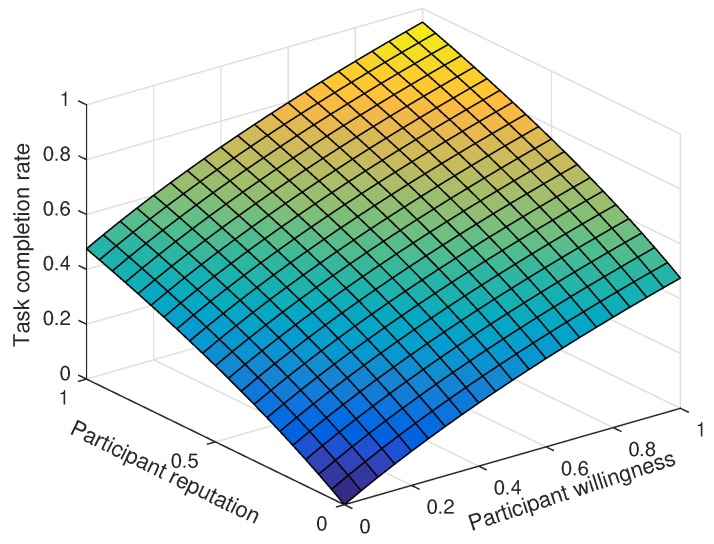
The influence of participant willingness and reputation on the task completion rate.

Due to the limited platform budget, only 10 of the 100 tasks are video tasks. As shown in Figure 4, both willingness and reputation positively contribute to the task completion rate. However, when a single characteristic attribute is at a high level, the task completion rate is much lower than that when both characteristic participant attributes are at high levels. When participant willingness and reputation are both 1, the task completion rate reaches 96%.

**Figure 5:**
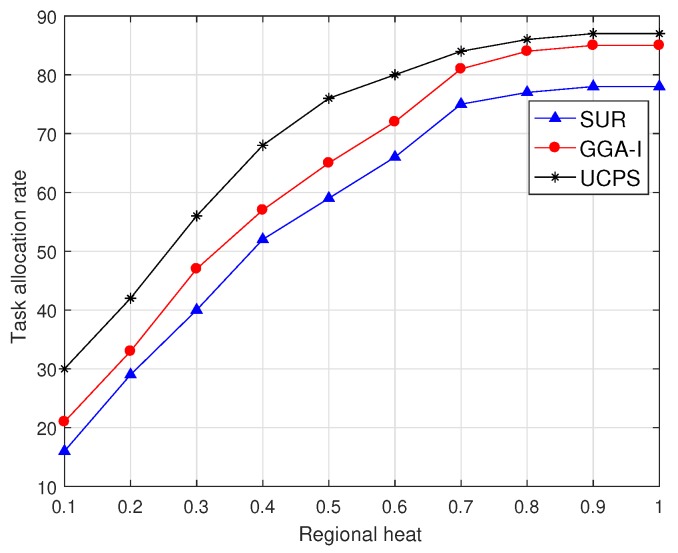
Task allocation rate under various regional heat.

Figure 5 shows that, when the service platform publishes 50 sensing tasks, the task allocation rate of the three algorithms present an upward trend with the increasing regional heat and the growth rate gradually decreases. In low-heat regions, the performance of the proposed UCPS-L is notably better than those of the other two algorithms because the community assistance strategy motivates other users to complete more allocated tasks. The SUR always selects the user with the highest data quality and only allocates a few tasks to low-heat regions, resulting in poor performance. When the regional heat is high, UCPS-H attracts more users to participate in the MCS tasks and has better performance than GGA-I. The allocation rate of SUR changes lightly after the regional heat exceeds 0.8. Because SUR always selects participants with the highest data quality without any payment constraints, more budgets are involved in the early stage of participant selection and the remaining budget is insufficient to select more participants, and therefore 17.4% of tasks cannot be allocated.

### 6.2. The Effect of Task Coverage Radius and Deadline on the Task Allocation Rate

**Figure 6:**
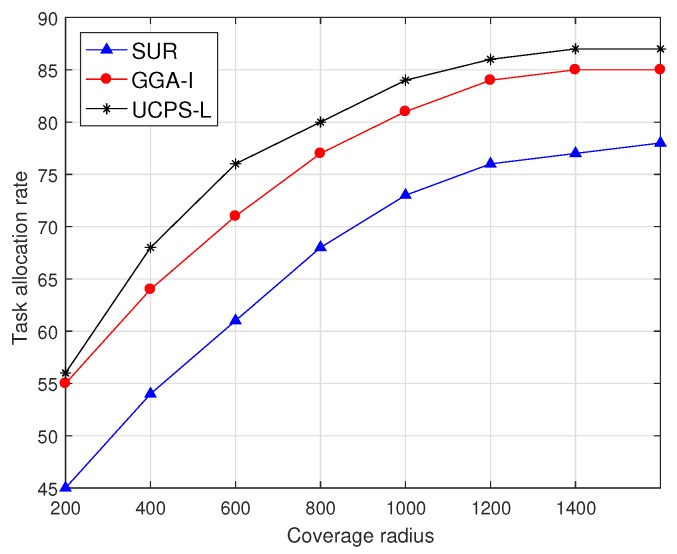
Task allocation rate under various task coverage radius.

As shown in Figure 6, the task allocation rate changes with the task coverage radius when the service platform publishes 50 sensing tasks for low-heat regions. The task allocation rate of SUR is always lower than those of the other two mechanisms because SUR only selects participants with the highest quality, and does not consider the situation where a single participant accomplishes multiple sensing tasks through storage and movements. In addition, because SUR always selects the participant with the highest quality and does not consider the user bid, the remaining budget is insufficient to select new participants and the task allocation rate is always lower than those of the other two algorithms. GGA-I selects the participant with the shortest moving distance to the task location and the participant can accept multiple sensing tasks, and therefore more tasks can be allocated to selected participants when the coverage radius is small. Similarly, participants in UCPS-L also accept multiple sensing tasks, and complete tasks through the community assistance strategy, which can allocate 86% of tasks when the coverage radius is 1200 m. When the coverage radius is more than 1400 m, the overhead for users to complete sensing tasks will increase dramatically; therefore, few users are willing to participate in sensing tasks.

**Figure 7:**
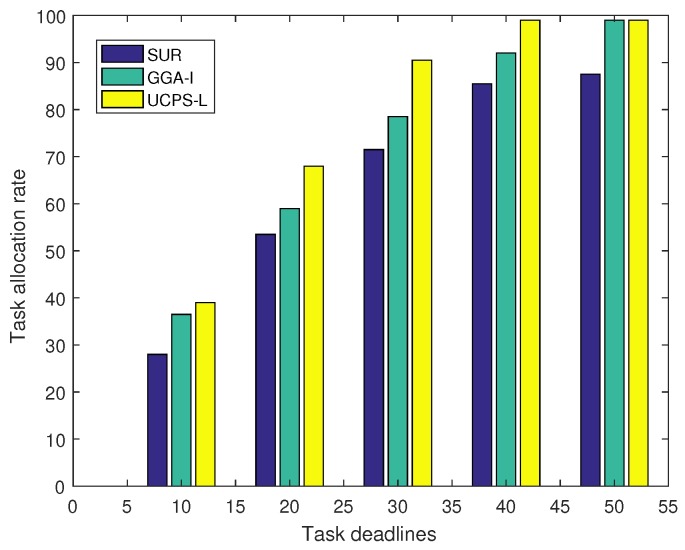
Task allocation rate under various deadlines.

Figure 7 illustrates the relationship between task deadlines and task allocation rates in low-heat regions and there are 50 sensing tasks published by the service platform. The longer deadline signifies more time for the participant to complete the task. As a result, the task allocation rate increases with the deadline. When the deadline is 10 min, the participant can only complete tasks that are close to him and the allocation rate is relatively low. With the increase of deadline in SUR, more users meet the requirements and the task allocation rate keeps growing until the budget is insufficient. In GGA-I, participants can complete more tasks due to task queuing. Similarly, in UCPS-L, participants use the queue mechanism to accept more tasks and the community assistance strategy to complete multiple tasks simultaneously. Thus, the UCPS-L algorithm can allocate 91% of tasks when the deadline is 30 min, shortening the time for receiving data reports.

### 6.3. Analysis of Average Task Completion Time and Service Platform Satisfaction

**Figure 8:**
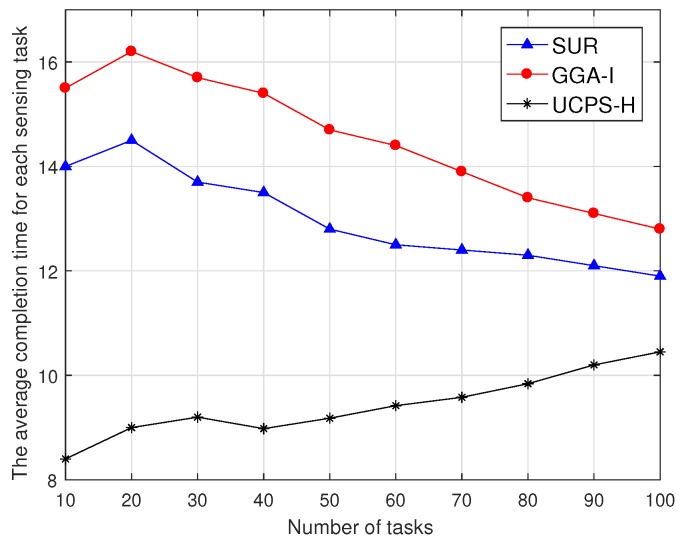
Comparison of average task completion time under total number of different tasks.

As shown in Figure 8, the average task completion time varies with the increasing total number of tasks in-high heat regions. The task completion time in this paper starts from accepting the task to uploading the data report to the service platform. When the number of tasks is small and the task sensing region is wide, SUR selects participants with the highest data quality from the user set, does not consider the time overhead, and increases the average task completion time. GGA-I selects participants with the shortest moving distance, but a single participant has to complete multiple sensing tasks, which also increases the average task completion time. With the increase in the number of tasks, tasks distributed within the region are more intensive and participants only select nearby tasks, and therefore the completion time is gradually reduced. UCPS-H considers the user willingness as an influence parameter on the comprehensive data quality, and selects nearby users to complete sensing tasks, which can reduce the time for completing sensing tasks. However, as the total number of sensing tasks increases, the number of potential participants per task decreases. To ensure the quality of service, the service platform has to wait for some busy participants to become idle and then participate in the sensing tasks again, and the task completion time increase.

**Figure 9:**
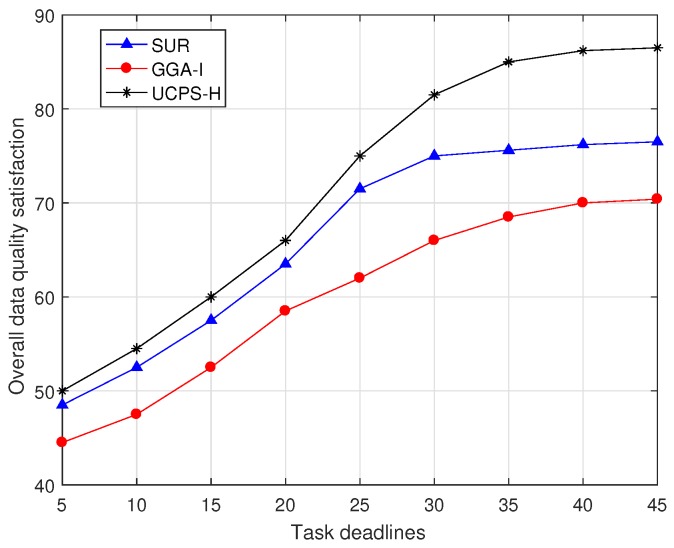
Comparison of data satisfaction at different deadlines.

Figure 9 illustrates the change in overall data quality satisfaction of the three algorithms as task deadlines increase in high-heat regions. The satisfaction in this paper is evaluated based on the number of tasks that meet the requirements. With the increase of task deadlines, GGA-I can obtain more sensing results and the satisfaction of the service platform is gradually increasing. However, its growing stops because GGA-I does not consider the data quality obtained by participants in completing tasks. As more sensing tasks are completed, task reports with poor data quality also increase, slowing the upward trend. However, SUR and UCPS-H allocate one task for each participant at a time and complete most of the tasks in a short time, so that the higher satisfaction can be achieved in the initial stage. For the 45-min task deadline, the satisfaction of UCPS-H is 13.1% and 22.7% higher than those of SUR and GGA-I.

## 7. Conclusions

In this paper, we have made an in-depth study on the problem of participant selection for MCS. We have proposed the User Characteristic aware Participant Selection mechanism for MCS, namely UCPS. Specifically, we estimate the regional heat according to the number of active users, average residence time of users and sensing tasks history. In addition, the user state information and sensing task records are combined to calculate the willingness, reputation and activity of users. Finally, the above four factors are comprehensively considered to reasonably select participants. We also propose task queuing and community assistance strategies to ensure task allocation rates and completion rates. The evaluation results have shown that UCPS has good performance, in terms of the task allocation rate, task completion rate, average task completion time, and data satisfaction. In future work, we will continue to study other factors affecting the participant selection in multi-task MCS scenarios and further analyze the relationship between user bids and data quality.

## Figures and Tables

**Table 1:** Scenario parameters.

Description	Value
Region size	1–2 km${}^{2}$
Regional heat	0–1
Task deadline	5–45 min
Number of participants required for single task	1–5
Number of tasks accepted by each participant	0–8
Number of tasks published by the service platform	1–100
Task coverage radius	200 m–1600 m
Participant reputation	0–1
Participant activity	0–1
Participant speed	10–50 km/h
Task types	5
The maximum budget for each sensing task	50
The maximum price per sensing task	10

## Appendix A

**Problem** **A1.** For a given task set ϑ and user subset U(U⊆N), the utility function $F_{U}\left(\vartheta \right)$ of the selected participant set U is a non-negative, non-reducing submodule.

**Proof.** It is straightforward that $F_{\emptyset }\left(\vartheta \right)=0,$ consider $N^{\prime }s$ two arbitrary subsets $U^{\prime }$ and *U*, $U^{\prime }\subseteq U\subseteq N$, we have $F_{U^{\prime }}\left(\vartheta \right)\leq F_{U}\left(\vartheta \right)$ (the equality hold if $K_{S^{\prime }-S=\emptyset }$), and hence $F_{U}\left(\vartheta \right)$ is nondecreasing. Consider that a participant u∈N−U, $\left|K_{U\cup \left\{u\right\}}\right|-\left|K_{U}\right|$ is the number of covers ϑ in $K_{u}$ that instead of $K_{U}$, then $\cup_{n_{j}\in U^{\prime }}K_{n_{j}}$ is at least as large as $\cup_{n_{j}\in U}K_{n_{j}}$. The utility function $F_{U}\left(\vartheta \right)$ is associated with the sum of the data quality obtained by ϑ in $K_{U}$, thus existing: $F_{U^{\prime }\cup \left\{u\right\}}\left(\vartheta \right)-F_{U^{\prime }}\left(\vartheta \right)\geq F_{U\cup \left\{u\right\}}\left(\vartheta \right)-F_{U}\left(\vartheta \right)$. In this rule, the difference in adding an element to the set $U^{\prime }$ is at least as great as the difference in adding the same element to the superset *U*. Thus, $F_{U}\left(\vartheta \right)$ is a monotone submodule. □
